# Aspects épidémiologiques, cliniques et prise en charge de sténose urétrale chez l’adulte dans un Hôpital de District de Ngaoundéré, Cameroun

**DOI:** 10.11604/pamj.2017.26.193.9669

**Published:** 2017-04-04

**Authors:** Ngaroua, Ngah Joseph Eloundou, Yaouba Djibrilla, Ousmane Asmaou, Amvene Jérémie Mbo

**Affiliations:** 1 Service de Chirurgie, Hôpital Régional de Ngaoundéré, Cameroun; 2 Département des Sciences Biomédicales, Université de Ngaoundéré, Cameroun; 3 Faculté de Médecine et des Sciences Biomédicales de l’Université de Yaoundé 1, Cameroun

**Keywords:** Sténose de l´urètre, prise en charge, Ngaoundéré, Urethral stenosis, management, Ngaoundere

## Abstract

**Introduction:**

L'objectif était de déterminer les aspects épidémiologiques, cliniques et prise en charge de la sténose de l’urètre à l’Hôpital Protestant de Ngaoundéré (HPN).

**Méthodes:**

Une étude rétrospective a été conduite, basée sur la revue des dossiers des patients hospitalisés pour sténose urétrale dans l’unité urologique de l’HPN, d’une période d’un an (janvier 2013 à janvier 2014). Seuls les dossiers complets étaient inclus.

**Résultats:**

57 patients ont été retenus sur les 69 dossiers enregistrés dont tous était de sexe masculin. L’âge moyen était 52,6 ans (19 ans à 85 ans). La tranche d’âge la plus représentée était celle de 41 à 60 ans. La sténose urétrale représentait 0,6% des consultations externes, 11,36 % des hospitalisations en chirurgie, et 6,96% des interventions chirurgicales dans cette structure sanitaire. La dysurie (70, 17%) était le principal motif de consultation. Les étiologies étaient d’origines infectieuses (52,63%), traumatiques (26,32%) et iatrogènes (21,05%). L’ECBU réalisé sur tous les patients a permis d’isoler les *gonocoques*(14,03%) et *Escherichia coli* (21,05%), le reste de culture était stérile (64,91%). L’Uretro-Cystographie Rétrograde (UCR) a été réalisée sur 28 patients (49,12%), dont 26 cas était des rétrécissements antérieurs (92,85%). L’Urétrotomie Interne Endoscopique (UTI) était le geste chirurgical le plus réalisé (58%). La sténose était le plus souvent localisée au niveau des régions bulbaires et péno-bulbaires. Le taux de guérison était de 87,73% contre 12,27% de récidives.

**Conclusion:**

La sténose urétrale est fréquente en Urologie de l’HPN mais les patients consultent souvent en stade compliqué. L’urétrotomie interne endoscopique a montré des résultats plausibles.

## Abstract (en)

**Introduction:**

The aim was to determine the epidemiological, clinical aspects and the management of these patients in Ngaoundere Protestant Hospital (HPN).

**Methods:**

We conducted a retrospective review of complete medical records of patients hospitalized for urethral stenosis in the Department of Urology at the Protestant Hospital of Ngaoundéré over a one-year period (January 2013 to January 2014).

**Results:**

Out of 69 registered medical records, a total of 57 patients files (all male) were identified for review. The average age was 52.6 (19 -85 years). The most represented age group was 41-60 years. Urethral stenosis accounted for 0.6% of consultations, 11.36 % of surgical hospitalizations and 6.96% of surgical procedures. Dysuria (70, 17%) was the main reason for consultation. Etiologies included infections (52,63%), traumas (26.32%) and iatrogenic problems (21.05%). All patients underwent CBEU which allowed to isolate gonococci (14,03%) and Escherichia coli (21.05%), the remainder of the urine culture was sterile (64.91%). 28 patients (49.12%) underwent retrograde uretrocystography (RUC), of whom 26 with anterior stenosis (92,85%). Internal endoscopic urethrotomy (IEU) was the most common surgical procedure (58%). Stenosis mainly occurred at the level of the bulbar and peno-bulbar regions. The rate of healing was 87,73% vs 12.27% of recurrences.

**Conclusion:**

Urethral stenosis is frequent in the Protestant Hospital of Ngaoundéré; patients requiring medical consultations are often in complex medical conditions. Internal endoscopic urethrotomy has shown plausible results.

## Introduction

Le rétrécissement urétral (ou sténose de l’urètre) est la réduction du calibre du canal de l’urètre, constituant un obstacle à l’écoulement normal des urines. Elle correspond à une réduction de calibre, plus ou moins étendue, du canal de l’urètre qui gêne le libre écoulement des urines de la vessie au dehors quel que soit son siège et son étiologie [1]. La lésion initiale est une rupture de la continuité urétrale en rapport avec une abrasion, une ulcération, une perforation, ou une dilacération de tout ou une partie de la paroi [2]. La particularité de cette affection réside sur son caractère récidivent et ses complications multiples. L’objectif de ce travail était de déterminer les aspects épidémiologiques, cliniques et prise en charge des sténoses de l’urètre à l’Hôpital Protestant de Ngaoundéré.

## Méthodes

Il s’agissait d’une étude rétrospective descriptive d’une période de douze mois (Janvier 2013-Janvier 2014), sur des patients au service d’urologie de l’Hôpital Protestant de Ngaoundéré, un hôpital de District de Santé au Cameroun qui compte environs 30 lits en chirurgie. Ont été inclus tous les dossiers médicaux des patients présentant un rétrécissement urétral, comportant des informations minimales suivantes: l’âge, le sexe, les antécédents, la notion de sondage vésicale et une observation médicale complète. Les données ont été analysées à l’aide du logiciel Epi info 7 selon la statistique courante. Par ailleurs, le test de Chi2 a été utilisé pour établir l’indépendance entre variables avec p<0,05.

## Résultats

**Figure 1:**
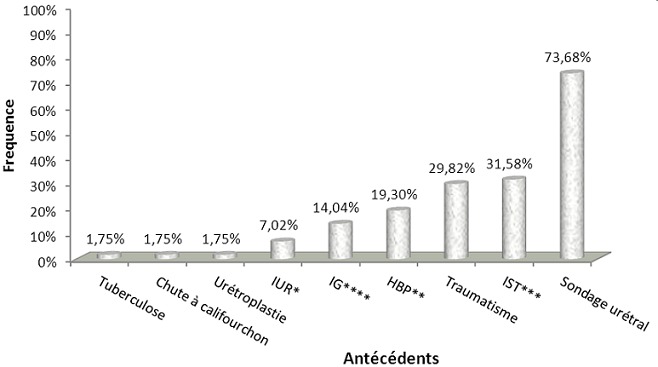
Distribution des différents facteurs favorisant la sténose urétrale chez les patients enregistrés (∗infections urinaires à répétitions; ∗∗hypertrophie bénigne de la prostate; ∗∗∗infection sexuellement transmissible; ∗∗∗∗infection gonococcique)

Sur 69 dossiers des malades enregistrés pour sténose urétrale, 57 ont été retenus. Tous les sujets étaient de sexe masculin (100%). L’âge moyen était de 52,6 ans (Ecart type=5) dont les extrêmes étaient de 19 et 85 ans. La tranche d’âge la plus représentée était celle de 41-60 ans. La majorité (70, 17%) a consulté pour dysurie alors que le reste (29,83%) était constitué de dysurie associée à d’autres symptômes tels que: la faiblesse du jet mictionnel, la pollakiurie, la nycturie, la fièvre, l’hématurie, la rétention aigue d’urine, et d’autres symptômes. En outre, la sténose urétrale a représentée 0,6% des consultations externes, 11,36% d’hospitalisation en chirurgie, et 6,96% des interventions chirurgicales durant cette période d’étude. Les antécédents de ces patients étaient pour la plupart les aux manœuvres vésicaux par sondage vesical suivis des infections sexuellement transmissibles et des traumatismes (Figure 1).

### Bilan para-clinique

L’Examen Cyto-Bactériologique des Urines était réalisé sur tous les patients. Les germes *gonocoques* (14,03%) et *Escherichia coli* (21,05%) étaient isolés, alors que 64,91% des résultats montraient une culture stérile, exempt des germes pathogènes. L’échographie était réalisée chez 12 patients (21,05%) et les résultats ont révélés une augmentation du volume de la prostate. L’Uretro-Cystographie Rétrograde (UCR) a été réalisée sur 28 patients (49,12%), dont 26 cas était constitués des rétrécissements antérieurs (92,85%). Ce qui traduit une exposition de la partie antérieur de l’urètre. De plus, 28,07% des sténoses étaient complexes contre 71,93% des sténoses simple.

### Prise en charge

L’Urétrotomie Interne Endoscopique (UTI) était le geste chirurgical le plus réalisé (58%) pour le traitement de sténose urétrale dans cette formation sanitaire. Tous les patients avaient reçu une antibiothérapie pendant une semaine puis, un traitement antalgique; une rééquilibration hydro électrolytique; un anti inflammatoire non stéroïdien et un sondage urétro-vésical d’une durée moyenne de 3 semaines. La région bulbaire était majoritairement représentée soit 49,12% suivi de la région péno-bulbaire 33,33%. La cause infectieuse était par ailleurs la plus fréquente avec un taux de 52,63%. Ces infections se localisaient beaucoup plus sur la région bulbaire et Peno-bulbaire. Il n’y a pas de relation statistiquement significative entre le siège et la cause (Tableau 1). La durée d’hospitalisation moyenne était de 4 jours, avec un seul cas d’infection de la paroi (1,8%). La guérison était enregistrées dans 87,73% des cas contre 12,27% de récidive.

**Tableau 1:** Répartition de sténose urétrale traitée en fonction du siège et de l'étiologie

Etiologies	Siege de la sténose urétrale				Total
	Bulbaire	Bulbo-membraneux	pénien	péno-bulbaire	
**Iatrogène**	8	0	1	3	12
**Infectieuse**	13	3	3	11	30
**Traumatique**	7	0	3	5	15
**TOTAL**	28	3	7	19	57

## Discussion

Nous avons recensé au service d’urologie de l’Hôpital Protestant de Ngaoundéré entre Janvier 2013 et Janvier 2014, 57 dossiers complets des patients souffrant de sténose urétrale répondant aux critères d’inclusion. Ceux-ci ont représenté 0,6% des consultations externes, 11,36% des hospitalisations au service de chirurgie et 6,96% des interventions chirurgicales. Ces résultats étaient comparable à ceux trouvé au Mali où la sténose urétrale représentait: 1,7% des consultations externes; 5,44% des hospitalisations et 5,56% des interventions chirurgicales; de même, 6,2% des hospitalisations dans un autre centre hospitalier de la même ville [3]. C’est une pathologie non rare en chirurgie qui survient à tout âge. Dans notre série, l’âge moyen des patients était de 52,6 ans avec un pic maximum dans la tranche 41-60 ans. De même, un résultat similaire a été trouvé en Côte d’Ivoire dans un service d’urologie dont l’âge moyenne était de 55 ans, mais avec un pic maximum entre 60-80 ans [4]; Dans une étude similaire au Togo, 95 patients avaient été enregistrés et l’âge moyen était de 44,71 ± 18 ans avec des extrêmes de 16 et 87 ans [5]. Et au Kenya, l’âge moyen était de 42,7 ans [6]. Ces résultats montrent que les adolescents et les adultes jeunes sont aussi concernés car constituent la population sexuellement active. Egalement la pluralité des partenaires, la liberté des mœurs favorisent les IST avec 31,56% d’antécédents d’IST dans notre étude. Les patients enregistrés étaient constituée à 100% des hommes comme retrouvée dans plusieurs études. Cette affection semble être rare chez le sujet féminin. Sur un échantillon de 112 patients enregistrés dans une étude rétrospective au Kenya, un seul sujet féminin a été enregistré [5].

### Clinique

La forme dysurique pure est la plus rencontrée dans les pays développés alors que dans notre contexte cette forme est associées à d’autre symptôme tels que la faiblesse de jet mictionnel (36,84%) et à la rétention d’urine à répétition (10,53%), expliquent le retard de consultation. Les étiologies des rétrécissements urétraux sont très variées. Au Sénégal, dans une étude rétrospective portant sur 414 rétrécissements urétraux, des étiologies infectieuses (63%), traumatique (13.7%) et iatrogène (8.2%) ont été retrouvés [7]. Dans notre série, les étiologies: Infectieuses (52,63%), traumatiques (26,32%) et iatrogènes (21,05%) étaient retrouvés avec 14,04% d’antécédent de gonococcique, et un seul cas de tuberculose urogénital. Ces origines infectieuses, traumatiques et iatrogènes sont par ailleurs retrouvées dans plusieurs études similaires [1, 7-9]. Ces résultats s’expliqueraient par la fréquence des infections urogénitales en milieu tropical, mais aussi le bas niveau socio-économique de la population, l’automédication, les multitudes accidents de voies publique et les consultations tardives. Les causes iatrogènes sont les plus souvent dues aux manœuvres endoscopiques [10]. Mais dans notre contexte, elle est due surtout aux sondages intempestifs.

### Prise en charge

Les rétrécissements étaient localisés majoritairement au niveau de l’urètre bulbaire dans 49,12% des cas et peno-bulbaire dans 33,33%, le reste intéressait la région pénienne et bulbo-membraneuse. Le siège bulbaire peut s’expliquer par la configuration du bulbe dont le cul-de sac constitue un réservoir où pullulent les germes du fait de la stase urinaire. C’est ce qui explique la localisation des rétrécissements d’origine infectieuse au niveau bulbaire.

### Techniques de traitement

Le traitement des sténoses urétrales était essentiellement chirurgical et plusieurs techniques ont été utilisées. L’Urétrotomie interne endoscopique (UTI) seul était la technique la plus réalisée dans le traitement des sténoses urétrales dans cette formation sanitaire (58%). C’est une technique applicable pour les sténoses courtes, uniques et franchissables à la lame pour l’urètre bulbaire [11]. Pourtant, elle a produit des résultats satisfaisant sur les sténoses dont la longueur allait jusqu´à deux centimètres. Les autres techniques étaient constituées de l’UTI associées à d’autres gestes tous ayant des bénéfices dans le traitement [12-14]. Plusieurs autres techniques sont en évolution mais, le plateau technique et le niveau économique de nos structures ne permettent pas de les réaliser.

### Evolution

Les suites opératoires ont été favorables avec 87,73% de réussite contre 12,27% de récidives. Des taux semblables étaient obtenus dans plusieurs études similaires [8, 15]. Par ailleurs, aucun cas de décès n’a été notifié.

## Conclusion

Le rétrécissement urétral est une pathologie très fréquente dans région. Les étiologies les plus fréquemment rencontrées étaient infectieuses et traumatiques. La cause iatrogène est non négligeable due aux manœuvres vésicales. Le traitement peut se faire en urgence ou en différé suivant le contexte. L’Urétrotomie interne endoscopique était la technique la plus utilisée. La prévention des infections sexuellement transmissible est une meilleure prévention primaire de cette affection.

### Etat des connaissances actuelle sur le sujet

- La sténose urétrale est une pathologie fréquente en urologie;
- Les manœuvres vésicales sont les principales étiologies;
- Ses complications sont redoutées.

### Contribution de notre étude à la connaissance

- La sténose urétrale est fréquent chez les hommes dans cette région;
- Les sondages urinaires, les infections sexuellement transmissibles et les traumatismes sont les principales étiologies;
- L’urétrotomie interne endoscopique est la technique produisant des bons résultats.
